# EXPERT: A practical and forward-looking guide to capturing protein expression and purification metadata to develop machine learning models

**DOI:** 10.1016/j.jbc.2026.113610

**Published:** 2026-09-29

**Authors:** Christopher D.O. Cooper, Evgeny Tankhilevich, Kim Remans, Ahmed El Marjou, Aled M. Edwards, Andrew R. Leach, Andrew Quigley, Anja Schütz, Anne Zemella, Ario de Marco, Åsa Sivertsson, Barbara Borgonovo, Ben Kemp, Bjørn Voldborg, Carlo Carolis, Carter Mitchell, Cecilia Wickstrand, Dafydd R. Owen, David Drechsel, David Jones, Diego A. Oyarzún, Dominic Esposito, Eric R. Geertsma, Ewa Krupinska, Frank Bernhard, Frederico Ferreira-da-Silva, Hanna Tegel, Ian Hunt, Imre Berger, James Love, Jan Dohnálek, Jelena Thies, Jerome Basquin, Jesse Coker, Johannes Buyel, Joop van den Heuvel, Jurgen Haustraete, Katharina L. Dürr, Kelvin Lau, Kristina Hedfalk, Kristof Bozovicar, Lihua Liu, Lisa Bamber, Luigi Angelo Scietti, Malin Bäckström, Maren Schubert, Mark Elvin, Martin Pelosse, Matthew H. Todd, Matthieu Schapira, Miroslava Alblová, Nicholas S. Berrow, Nikolay Marinchev Dobrev, Oleg Brodsky, Ondřej Vaněk, Opher Gileadi, Pam Dossang, Paola Storici, Patrick Celie, Paul Wan, Peter A. Loppnau, Rachel J. Harding, Ray Owens, Renaud Vincentelli, Richard Altman, Rick Davies, Rob Meijers, Robbert Kim, Ruth Saxl, Sabine Suppmann, Sameer Velankar, Sebastiano Pasqualato, Sergio Martinez Cuesta, Stéphane Petres, Steven Harborne, Susanne Witt, Svend Kjaer, Tamar Unger, Timothy Craig, Tsafi Danieli, Ugis Sarkans, Wolfgang Knecht, Wolfgang Kuttenlochner, Yoav Peleg, Lovisa Holmberg Schiavone, Nicola A. Burgess-Brown

**Affiliations:** 1 School of Biosciences, Faculty of Health and Medical Sciences, University of Surrey, Guildford, United Kingdom; 2 European Molecular Biology Laboratory, European Bioinformatics Institute (EMBL-EBI), Hinxton, United Kingdom; 3 European Molecular Biology Laboratory, Protein Expression and Purification Core Facility, Heidelberg, Germany; 4 Institut Curie, CurieCoreTech, PSL University, Recombinant Proteins Core Facility, CNRS, Paris, France; 5 Structural Genomics Consortium, University of Toronto and University Health Network, Toronto, Ontario, Canada; 6 Membrane Protein Laboratory, Research Complex at Harwell, Diamond Light Source, Didcot, United Kingdom; 7 Max-Delbrück-Center for Molecular Medicine in the Helmholtz Association (MDC), Technology Platform for Protein Production & Characterization, Berlin, Germany; 8 Fraunhofer Institute for Cell Therapy and Immunology, Branch Bioanalytics and Bioprocesses (IZI-BB), Potsdam, Germany; 9 Laboratory of Environmental and Life Science, University of Nova Gorica, Nova Gorica, Slovenia; 10 Department of Protein Science, SciLifeLab, KTH-Royal Institute of Technology, Stockholm, Sweden; 11 Max Planck Institute of Molecular Cell Biology and Genetics, Protein Biochemistry facility, Dresden, Germany; 12 Biomodal, Chesterford Research Park, Cambridge, United Kingdom; 13 Department of Biotechnology and Biomedicine, Technical University of Denmark, Lyngby, Denmark; 14 Centre for Genomic Regulation (CRG), The Barcelona Institute of Science and Technology, Barcelona, Spain; 15 Kemp Proteins, LLC, Frederick, Maryland, USA; 16 Mammalian Protein Expression Core Facility and Protein Production Sweden, Sahlgrenska Academy, University of Gothenburg, Gothenburg, Sweden; 17 Pfizer Research and Development, Cambridge, USA; 18 Vienna BioCenter Core Facilities GmbH, Vienna, Austria; 19 University College London, Department of Computer Science and Department of Structural and Molecular Biology, London, United Kingdom; 20 Generative Biology Institute, Ellison Institute of Technology Oxford, The Oxford Science Park, Oxford, United Kingdom; 21 School of Informatics, The University of Edinburgh, Edinburgh, United Kingdom; 22 School of Biological Sciences, The University of Edinburgh, Edinburgh, United Kingdom; 23 Septerna, South San Francisco, USA; 24 Lund Protein Production Platform & Protein Production Sweden & Science for Life Laboratory, Lund University, Lund, Sweden; 25 Institute of Biophysical Chemistry, Goethe-University Frankfurt, Frankfurt/Main, Germany; 26 i3s - Instituto de Investigação e Inovação em Saúde, Universidade do Porto, Porto, Portugal; 27 IBMC - Instituto de Biologia Molecular e Celular, Universidade do Porto, Porto, Portugal; 28 Novartis, Biomedical Research, Cambridge, Massachusetts, USA; 29 Max Planck-Bristol Centre for Minimal Biology, Schools of Chemistry and Biochemistry, University of Bristol, Bristol, United Kingdom; 30 Lila Sciences, Cambridge, USA; 31 Institute of Biotechnology of the Czech Academy of Sciences, Laboratory of Structure and Function of Biomolecules, Vestec, Czech Republic; 32 School of Pharmacy and Structural Genomics Consortium, UCL, London, United Kingdom; 33 Max Planck Institute of Biochemistry, Protein Production and Structural Validation Facility, Martinsried, Germany; 34 Cleveland Clinic Center for Therapeutics Discovery, Cleveland Clinic Research, Cleveland, USA; 35 BOKU University, Department of Biotechnology and Food Science (BTLW), Institute of Bioprocess Science and Engineering (IBSE), Vienna, Austria; 36 Helmholtz Centre for Infection Research, Department of Molecular Structural Biology (MOSB), Braunschweig, Germany; 37 VIB Protein Core, VIB Technologies, VIB, Ghent, Belgium; 38 Department of Biomedical Molecular Biology, Ghent University, Ghent, Belgium; 39 Department of Chemistry, Kavli Institute of Nanoscience Discovery, University of Oxford, Oxford, United Kingdom; 40 Protein Production and Structure Characterization Core Facility (PTPSP), School of Life Sciences, École Polytechnique Fédérale de Lausanne (EPFL), Lausanne, Switzerland; 41 Department of Chemistry and Molecular Biology, Biochemistry, University of Gothenburg, Gothenburg, Sweden; 42 Abcam, Discovery Drive, Cambridge Biomedical Campus, Cambridge, United Kingdom; 43 Biochemistry and Structural Biology Unit, Department of Experimental Oncology, IRCCS European Institute of Oncology, Milan, Italy; 44 Virus-Like-Particle Based Technologies, Helmholtz Centre for Infection Research, Braunschweig, Germany; 45 Sygnature Discovery, Nottingham, United Kingdom; 46 European Molecular Biology Laboratory, Grenoble, France; 47 Protein Expression Core Facility, Institute for Research in Biomedicine (IRB Barcelona), The Barcelona Institute of Science and Technology, Barcelona, Spain; 48 Data Powered Therapeutics GmbH, Annastraße 27, Göttingen, Germany; 49 Medicine Design, Pfizer Worldwide R&D, San Diego, California, USA; 50 Department of Biochemistry, Faculty of Science, Charles University, Prague, Czech Republic; 51 Center for Molecular Medicine, Karolinska Institutet, Stockholm, Sweden; 52 Independent Consultant, United Kingdom; 53 Protein Targets for Drug Discovery Lab, Structural Biology, Elettra Sincrotrone Trieste, Basovizza, Trieste, Italy; 54 NKI Protein facility, Division of Biochemistry, The Netherlands Cancer Institute (NKI), CX, Amsterdam, The Netherlands; 55 Viva Biotech, Shanghai, China; 56 The Rosalind Franklin Institute, Harwell Science Campus, Didcot, United Kingdom; 57 Division of Structural Biology, Nuffield Department of Medicine, University of Oxford, Oxford, United Kingdom; 58 AFMB UMR 7257 CNRS-AMU, Marseille, France; 59 Thomson Instrument Company, Carlsbad, California, USA; 60 Institute for Protein Innovation, Neuroscience Program, Boston, Massachusetts, USA; 61 Department of Cell and Chemical Biology (CCB), Leiden University Medical Centre (LUMC), Leiden, The Netherlands; 62 The Jackson Laboratory, Protein Science Services, Maine, USA; 63 Fraunhofer Institute for Translational Medicine and Pharmacology ITMP, Immunology, Infection and Pandemic Research IIP, Penzberg, Germany; 64 Human Technopole, Milan, Italy; 65 Data Sciences and Quantitative Biology, Discovery Sciences, R&D BioPharmaceuticals, AstraZeneca, Cambridge, United Kingdom; 66 Institut Pasteur, Université Paris Cité, Production and Purification of recombinant Proteins core facility, CNRS UMR3528, Paris, France; 67 Protein Production Core Facility, University Medical Centre Hamburg Eppendorf, Hamburg, Germany; 68 Structural Biology STP, Francis Crick Institute, London; 69 Life Sciences Core Facilities (LSCF), Structural Proteomics Unit (SPU), Weizmann Institute of Science, Rehovot, Israel; 70 Medicine Design, Pfizer Worldwide R&D, Groton, Connecticut, USA; 71 Protein Expression Facility, Alexander Silberman Institute of Life Sciences, The Hebrew University of Jerusalem, Jerusalem, Israel; 72 Protein Sciences, Structure and Biophysics, Discovery Sciences, R&D, AstraZeneca, Gothenburg, Sweden

**Keywords:** Protein expression, Protein purification, Heterologous protein production, Machine learning, Predictive modelling, Protein engineering, Bioprocess optimisation, Reproducibility, Data standardization, Metadata framework, FAIR data

## Abstract

As machine learning (ML) becomes increasingly important in protein engineering, synthetic biology, and bioprocess optimization, the lack of consistent, complete, and standardized experimental reporting remains a major barrier to building effective models. **EXPERT** (**EXpression and Purification Experimental Reporting Template**) defines a structured framework for capturing protein expression and purification data across the community, to increase reproducibility across laboratories and support downstream ML applications. We propose here the essential data that should be recorded during expression and purification workflows by both novice and experienced protein production scientists. These recommendations are informed by expert contributions from across academia and industry. The reporting requirements were distilled to a focused set of parameters deemed sufficient for workflow reproducibility and constructing robust ML models aimed at improving protein production. By deploying these guidelines, we hope to enable the collection of larger data sets to support future model building.

Despite decades of methodological development, heterologous protein production remains challenging, particularly in achieving sufficient yield and quality, solubility, and functional activity. Researchers increasingly focus on more complex, aggregation-prone, or otherwise recalcitrant proteins. These proteins, such as membrane, intrinsically disordered, and large multi-component complexes, remain underrepresented in the literature despite their relevance as drug targets 1, 2, 3, 4. Across the global research community, many laboratories repeatedly attempt to produce the same proteins, often unsuccessfully, resulting in duplicated trial-and-error efforts and substantial costs in time, funding, and resources (5). These issues of reproducibility could be mitigated by creating an openly available data resource detailing the protocols and the outcomes of protein production experiments. Knowledge transfer could be further facilitated by using such a resource alongside academic publication.

Advances in artificial intelligence (AI), particularly machine learning (ML) are rapidly transforming the life sciences, offering new opportunities to address persistent challenges in protein production and engineering 6, 7, 8. Developing ML models to predict expression and purification success in specific host systems under defined conditions would reduce empirical screening, accelerate construct design, and shorten the timeline from digital design to purified protein for downstream applications. The success of AI models for protein structure prediction illustrates the transformative impact of combining computational methods with large, standardized, community-curated datasets (6). This breakthrough was enabled by decades of systematic deposition of structural data into the Protein Data Bank (PDB) 9, 10 and rigorous benchmarking efforts carried out through the Critical Assessment of Protein Structure Prediction (CASP) initiative (11). By analogy, a similar advance in predictive protein production will require the development of large, diverse and high-quality datasets that capture the key experimental variables governing expression and purification outcomes. Such data standardisation initiatives have precedent amongst stakeholder communities, with MIAME (Minimum Information About a Microarray Experiment) (12), a well-known example where data deposition into public microarray repositories became both highly recommended by users and widely adopted by scientific publishers (13).

The concept of systematically capturing experimental results from recombinant protein production in a standardized format to enable metadata-driven analysis led to the Minimal Information for Protein Functional Evaluation (MIPFE) initiative (14). At the time of their introduction, these proposals failed to gain traction, primarily due to the significant technical and logistical challenges associated with constructing such datasets. Protein production workflows involve many interdependent variables, such as construct design, host strain, growth and induction conditions, and purification strategy, that are often inconsistently reported 1, 2, 3. Technical variability in heterologous systems further limits reproducibility, with the same construct yielding different results across or even within laboratories 5, 15. Quality-control methods also vary widely, from sodium dodecyl sulfate-polyacrylamide gel electrophoresis (SDS-PAGE) to chromatographic and functional assays, complicating standardization (5). With growing impetus to integrate such multiparametric and heterogeneous datasets into ML models, coupled with increasing acceptance of experimental irreproducibility, there is now broad community engagement in the development of such frameworks for systematic data capture.

The complexity of protein expression and purification workflows frequently results in incomplete records, as practices vary widely, from electronic lab notebooks to bespoke databases and informal documentation, with minimal harmonization. Even basic measurements, such as protein quantification, frequently lack sufficient methodological detail. Differences in assays (*e*.*g*., Bradford, BCA, UV absorbance), and assumptions such as extinction coefficients are often omitted, limiting reproducibility and cross-study integration. In parallel to the establishment of a robust reporting framework, there is also a need to create a public resource to host protein production data and to make it accessible to the community. Established resources such as UniProtKB and the PDB host protein nomenclature, structural, and functional data. The European Molecular Biology Laboratory-European Bioinformatics Institute (EMBL-EBI) BioStudies database provides a suitable platform for storing protein production experimental results and can be further refined as additional data becomes available.

Although substantial datasets exist for certain well-studied targets, particularly human proteins, the overall dataset lacks the diversity needed to support generalizable predictive models. Many protein classes, hosts and expression contexts remain underrepresented, constraining the development of models that perform robustly across biological systems. Furthermore, some published data may be misleading due to the lack of verification of biological materials, with vector and insert sequences occasionally differing from those reported. Uncertainty also persists regarding which data should be captured today that would support future ML applications beyond protein production, risking the loss of valuable contextual information. Existing data are heavily skewed towards protein engineering, antibody production, and *Escherichia* *coli*-based expression systems, with comparatively limited focus on general protein production workflows. Moreover, there remains a lack of meaningful translation of these approaches to the challenging target classes. Compounding these issues, methodological details in biochemical and structural biology publications are frequently insufficient or inconsistent, making experimental reproduction difficult or impossible. Together, these challenges underscore the need for coordinated data standards, clearer guidance on metadata capture, and a systematic approach to data sharing. Addressing these issues is essential for enabling reproducible protein production and for building robust, generalizable predictive models.

The systematic omission of negative data (meaning data that behave similarly to a negative control) represents a particularly significant limitation. Failed expression constructs, insoluble products and unsuccessful purification attempts are rarely documented, yet these outcomes are essential for defining the boundaries of success and failure in method development and in the development of ML models, and for avoiding biased or overly optimistic predictions. Negative data should therefore be collected with the same care as “positive” data and included in the experimental datasets reported.

To support the development of robust ML models for predictive protein production, the field requires a standardized framework that identifies and prioritizes the essential metadata necessary for reproducible, machine-usable datasets. The creation of such frameworks aligns with community efforts to promote FAIR (Findable, Accessible, Interoperable, Reusable) data principles (16) and broader initiatives aimed at improving data interoperability in the biosciences (17). In this context, we present EXPERT (EXpression and Purification Experimental Reporting Template); a consensus-driven set of reporting criteria, developed in collaboration with experts across the protein science community.

## Aim and objectives

To address these challenges in recombinant protein production and reproducibility, we propose to develop an advisory framework for minimal yet essential data collection in protein expression and purification experiments. The goal of this framework is to enhance inter-laboratory reproducibility while enabling the generation of high-quality, standardized datasets. Such datasets will serve as a foundation for building predictive models, ultimately advancing data-driven and predictive approaches to protein production.

Benchmarking initiatives built on top of these datasets would be particularly valuable for assessing the robustness of the proposed guidelines across different experimental contexts, including protein classes, expression systems, vector architectures, and fusion tags. Such efforts could also be developed in collaboration with publishers, establishing common standards for annotation of academic papers.

We hope that, in the future, this work will contribute to the establishment of a systematic approach for achieving reliable protein production with sufficient yield, solubility, and, where required, functional activity. This includes the selection of an appropriate expression system and the rational design of expression constructs tailored to the target protein’s properties and subsequent applications.

## What has been done so far in this field?

Efforts for data standardization have been established in allied fields, including choosing a suitable expression system and quality control applied to protein production 18, 19, 20. The Protein Structure Initiative (PSI) was a high-throughput structural genomics program that operated from 2000 to 2015. During this period, extensive protein production data were collected and ultimately deposited in the TargetTrack database (https://doi.org/10.5281/zenodo.821653). Data capture ended in 2017 when operations ceased. A subset of TargetTrack was later used to develop NetSolP, a predictive model for protein solubility and usability in *E. coli* (21).

Another group applied computational biology and machine learning to data from the Human Protein Atlas to identify protein fragments suitable for expression in *E. coli* for antibody production. Their analysis suggested that several intrinsic properties, including hydrophobicity, charge, aromaticity, and secondary structure, can already help guide the design of more successful expression experiments (22).

More recently, attention turned to protein secretion from eukaryotic systems, particularly Chinese Hamster Ovary (CHO) cells. One study analysed more than 2000 human protein sequences using 218 biochemical and biophysical descriptors. They identified intrinsic sequence features that explain approximately 15% of the variability in secretion efficiency. Key determinants included molecular weight, cysteine content, and extent of N-linked glycosylation. This work established a roadmap for the rational design of difficult-to-express proteins (23). RP3Net, an AI model for predicting small-scale heterologous soluble protein expression in *E. coli*, was recently developed in collaboration between AstraZeneca (AZ) and EMBL-EBI (24). This work leveraged open data from the Structural Genomics Consortium (SGC, Fig. 1) and proprietary data from AZ. During prospective experimental validation, RP3Net has outperformed other existing models, reporting accurate results for 77% of the constructs, and correctly identifying 92% of successfully produced constructs.

**Figure 1:**
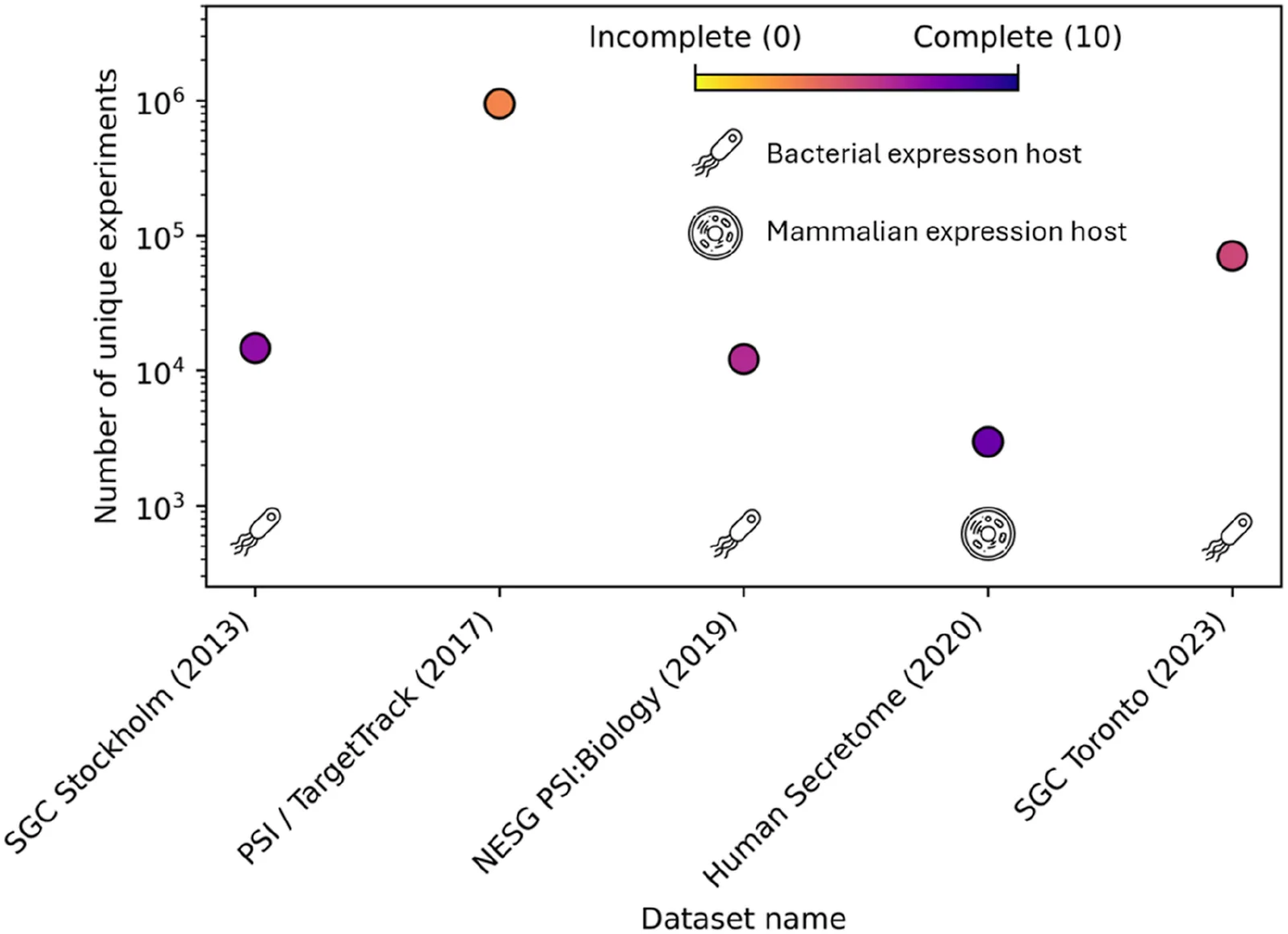
**Existing protein production datasets.** The recently published datasets PSI, Northeast Structural Genomics Consortium (NESG) PSI:Biology, Structural Genomics Consortium (SGC) Stockholm, Human Secretome and Structural Genomics Consortium (SGC) Toronto are ordered chronologically. Where the expression host species is known, the datasets are marked with an icon (bacterial, mammalian). PSI/TargetTrack data was collected from multiple host species. The marker colour indicates the completeness of the datasets, with respect to the metadata in Supplementary Sheet S2. More details, including the full citations, are provided in Supplementary Table S1.

In 2025, the Align Foundation published a review in which they concluded that the primary bottleneck to progress is the lack of large, high-quality datasets (20). Such initiative led to the recent development of a HiBiT luminescence-based reporter as a scalable and quantitative approach to measure protein expression across hosts (https://doi.org/10.5281/zenodo.19489506), which demonstrates the potential impact of community-led campaigns for large-scale data acquisition.

Described in Table 1 is a phased roadmap toward predictive protein production which proceeds in phases, aligned with data availability and model maturity.

**Table 1:** A phased Roadmap Toward Predictive Protein Production

Phase	Focus	Key Activities	Expected outcome
Near Term 1–3 years	Establishing the foundations	Implement EXPERT as a community-endorsed data standard with controlled vocabularies: • Publicise the EXPERT framework at scientific conferences/meetings. • Engage with publishers to implement the EXPERT framework for annotating academic publications that involve protein production. • Capture quality data from new experiments prospectively. • Build a user-friendly database to host community depositions and allow access for verifying experimental reproducibility and AI/ML model development. • Prioritise a limited number of expression systems to test predictions.	A consistent, accessible, and growing dataset that enables reproducible analysis and the development of early predictive models.
Medium Term 3–5 years	Expanding data and model capability	• Develop class-specific or modular predictive models (following DOME recommendations for instance (30)). • Integrate legacy datasets where curation effort is justified. • Incorporate structural and biophysical predictions as auxiliary model inputs. • Include other expression systems. • Organise benchmarking initiatives aimed at validating the hypotheses inferred by data analysis.	More capable and better-validated models, supported by broader datasets and improved integration of experimental and computational information.
Medium-long Term 5+ years	Toward generalisable prediction	• Develop predictive models capable of generalising across protein classes, diverse structural folds, and different expression hosts. • Establish continuous learning frameworks that update predictions as new data are deposited. • Enable broader reuse of protein production data for applications beyond expression and purification, including protein engineering and functional annotation (20).	A continuously improving predictive ecosystem that supports protein production across diverse biological contexts and enables new applications of the data.

Together, these phases provide a realistic pathway from standardized data capture and community deposition toward increasingly generalisable, continuously improving models for predictive protein production.

## Enabling adoption and sustainability

For this roadmap to succeed, data standards must remain pragmatic, extensible and fit-for-purpose, avoiding barriers to adoption while remaining future-proof. Alignment with trusted international data infrastructures and repositories will be critical for long-term sustainability, versioning of standards, and community governance. Linking data standards to a phased modelling roadmap can shift protein science from fragmented experimentation to a coordinated, data-driven ecosystem, where every dataset advances both individual projects and predictive protein production overall. This has obvious advantages both for data-sharing across organizations but also within organizations to maintain internal consistency, reproducibility, and reporting of data.

## Actions to improve protein production data capture

Addressing the challenges in protein production data capture requires coordinated action from the protein science community within both academia and industry. Below, we outline a set of practical and scalable measures that can significantly improve data quality, reproducibility and reusability, while enabling the development of predictive models.

## Establish a community advisory framework

A central step is the development of a consensus-based advisory framework that defines which experimental parameters should be captured during protein expression and purification. This framework should be designed by protein scientists, for protein scientists, with a focus on practicality and broad adoption. For example, a protein community advisory group could be created within existing frameworks of ELIXIR (https://elixir-europe.org/) or Protein Production and Purification Partnership in Europe (P4EU) (https://p4eu.org/).

To minimize burden while maximizing value, parameters should be organized into a prioritized hierarchy, such as:

• Critical parameters (essential for interpretation and reproducibility),
• Highly enabling parameters (substantially improve data utility),
• Optional or “nice-to-have” parameters.

Separate but harmonized parameter sets should be defined for protein expression and for large-scale protein purification, potentially summarized in tabular form for ease of use.

## Define and standardize negative data

Clear definitions of negative and positive outcomes are essential, particularly for downstream ML applications. The framework should specify what constitutes negative data for:

• Small-scale expression screening (*e*.*g*., no sign of expression, low yield due to high background contamination or affinity-purified protein by SDS-PAGE),
• Scale-up expression (as above, though protein is only likely to proceed to large-scale expression if small-scale readout was successful),
• Purification workflows (*e*.*g*., aggregated protein by Size Exclusion Chromatography (SEC) or degraded protein by SDS-PAGE or mass spectrometry (MS)).

This includes setting quantitative or semi-quantitative thresholds for common readouts (*e*.*g*., expression levels, solubility, yield, purity, monodispersity). Standardizing these definitions will ensure consistency across laboratories and enable more reliable model training. While future models may incorporate continuous rather than binary outcomes, well-defined thresholds remain critical in the near term.

## Capture data across expression systems

Data capture should explicitly account for the diversity of commonly used expression systems, including:

1. Bacterial systems (*E*. *coli*-based),
2. Insect cell systems,
3. Mammalian cell systems,
4. Cell-free systems.

For each system, the framework should recommend:

• A core set of expression parameters to record,
• Guidance on distinguishing successful from unsuccessful outcomes,
• Harmonization where possible to enable cross-system comparisons.

Similar principles should be applied to scale-up and purification workflows, with system-specific considerations where necessary.

## Standardize purification and downstream characterization

A limited and consistent set of parameters should be defined for protein purification, regardless of expression system. This should include clear criteria for identifying unsuccessful purification outcomes. Chromatographic settings should be described in sufficient detail to enable reproducibility (*e*.*g*., the specific resin used, buffer conditions, *etc*.), and we propose a standardized buffer component and concentration structure rather than free text to facilitate data entry and mining (Table 2 and Supplementary Sheet S2).

**Table 2:** Fragment of the EXPERT Framework Illustrating the Bacterial Expression System

Metadata field	Description of data	What to capture	Importance
Target			
T1	Protein name (designed construct)	Protein name according to UniProt naming convention, along with construct boundaries and tags	Critical
T2	Gene name (HGNC)	Gene name according to HGNC naming convention	Highly enabling
T3	Gene species (NCBI)	Taxonomy according to NCBI	Optional
Construct			
C1	DNA sequence of coding region including tag sequences	Nucleotide sequence from start ATG to stop codon. Should be the sequence that is used for expression including any DNA codon optimisation	Critical
C2	Amino acid sequence including tag sequences	Amino acid sequence from start methionine to last amino acid	Critical
Expression			
E1	Host strain/Cell line	Name of *E. coli* strain used (*e*.*g*., BL21 (DE3)).	Critical
E2	Culture medium	Name and composition of medium used (*e*.*g*., TB)	Critical
E4	Culture conditions – temperature post-induction	Temperature used post-induction (*e*.*g*., 18 °C)	Critical
EB1	Culture conditions -temperature pre-induction	Temperature used pre-induction (*e*.*g*., 37 °C)	Critical, bacterial-specific
E11	Lysis buffer	Formatted as BUFFER string in separate tab in Supplementary Sheet S2	Highly enabling
Purification			
P1	First-step purification method	Type of purification (*e*.*g*., Ni-NTA)	Critical
P2	First-step purification buffer	Formatted as BUFFER string in separate tab in Supplementary Sheet S2	Highly enabling
Quality			
Q1	Final batch - purity	How pure is the protein as determined by SDS-PAGE - need to comment which purification step purity has been assessed after	Highly enabling
Other			
O1	Other comments	Any other information deemed useful to supplement the deposition	Optional

The complete framework, including detailed information for all expression systems, is provided in Supplementary Sheet S2. A graphical overview of EXPERT is shown in Figure 2.

Downstream biophysical and biochemical characterization methods (*e*.*g*., assessments of purity, oligomeric state, stability, and functionality) should also be incorporated. While negative data may be less common at this stage, meaningful thresholds should still be established to ensure interpretability and comparability.

## Enable data sharing and sustainable infrastructure

To support large-scale model building, protein scientists should be encouraged to openly share protein production data whenever possible. This will require both cultural change and technical infrastructure.

A sustainable, trusted repository for protein production data should be identified or developed. Existing international bioinformatics infrastructures, such as those hosted by EMBL-EBI, represent a potential model for long-term data stewardship. Partnerships with established resources, such as the PDB, particularly for proteins produced for structural biology, could further inform minimal reporting standards and data integration strategies.

## Engage publishers and protect confidentiality

Journal publishers play a critical role in improving data quality and reproducibility. Encouraging journals to adopt more stringent requirements for reporting protein production methods and quality control metrics would significantly raise standards across the field. At the same time, mechanisms must be established to support confidential or anonymized data capture, as protecting sensitive information, such as proprietary targets, will be essential for broad engagement while still allowing valuable methodological data to be shared.

## What data parameters to capture?

Figure 2 provides an overview of the proposed EXPERT framework and what data should be captured. The detailed lists of the parameters to capture for different expression systems, along with descriptions, formats and importance levels, alongside a proposed format to record purification buffer composition, are available in the data capture Excel sheets (Supplementary Sheet S2). An excerpt from this metadata for bacterial systems is shown in Table 2. The data model contains intentional redundancies, allowing for information duplication. For example, when multiple constructs are generated using the same protocol, the fields describing expression and purification conditions will be identical. The framework is intended to provide sufficient flexibility to incorporate additional metadata fields in future versions, for example, to support more granular data capture or accommodate the development of new experimental procedures.

**Figure 2:**
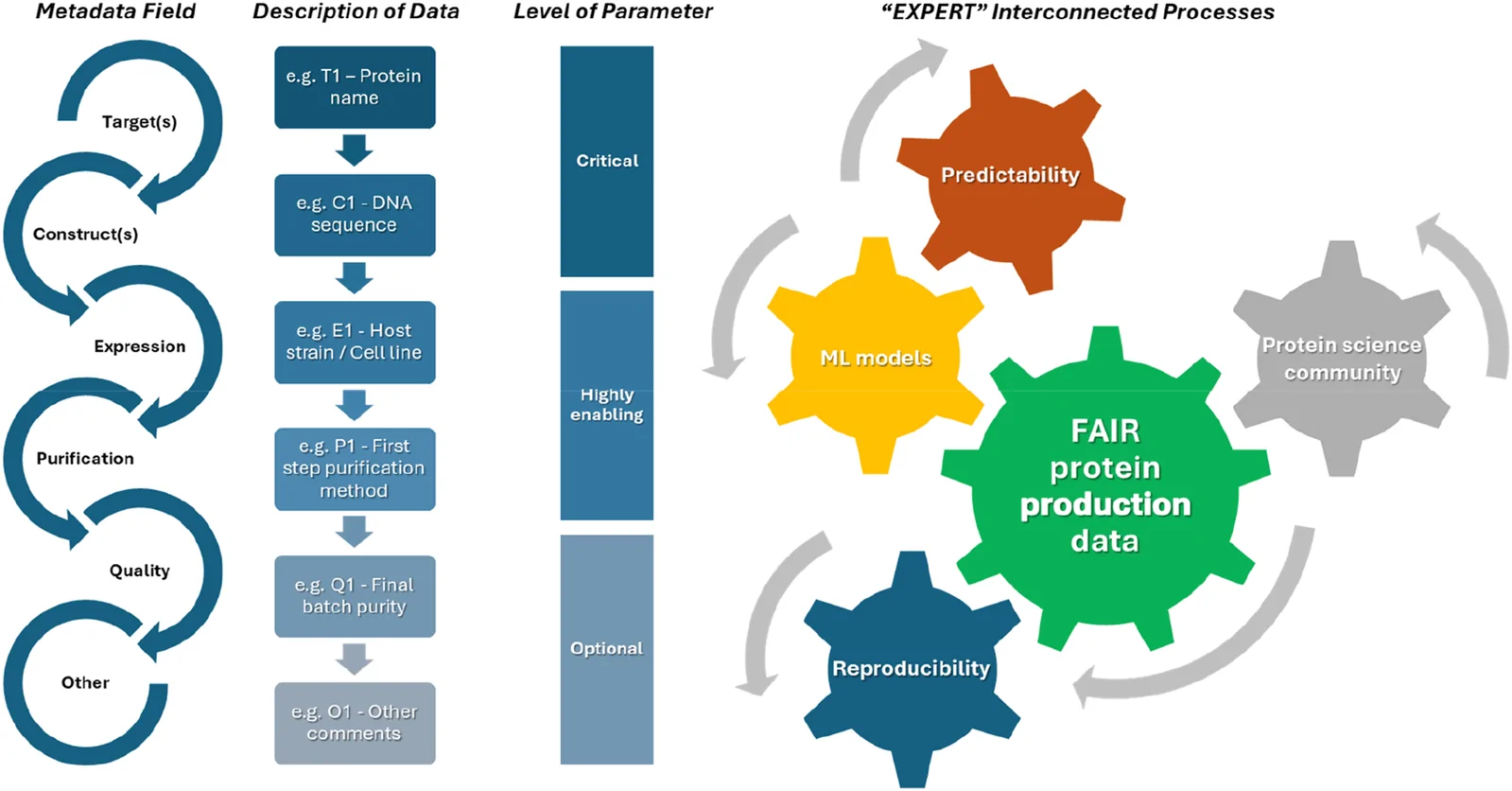
**Overview of proposed framework and data to capture.** The EXPERT Framework outlines the metadata that should be collected, describes the data and the importance of each parameter (critical, highly enabling, or optional), and provides an overview of how the different components are linked.

In the initial version of this framework, we propose using spreadsheets for data exchange and deposition. This approach may evolve as the templates gain adoption within the scientific community, potentially leading to the development of a more sophisticated user interface and an Application Programming Interface (API).

Within each field, the metadata specifies the field identifier, name, a description of the information to be captured, and its importance level. The latter serves only as guidance for depositors; we do not propose rejecting submissions due to missing critical fields. However, we encourage assigning a lower completeness score to incomplete submissions (Supplementary Sheet S2, Field O2).

Each data record captures information about the target, construct, protocol details, and the outcomes of gene expression, protein purification, and quality control. The proposed data format supports both single proteins and multimeric complexes. For complexes, multiple rows describing the production of individual components are linked by a common identifier. Each attempt to produce a complex under new conditions is assigned a new identifier, duplicating all component entries, including those that remain unchanged. This approach accommodates a range of complex formation strategies, including, but not limited to, polycistronic constructs, co-expression from multiple vectors, and separately purified components.

The supplementary data capture spreadsheet includes dedicated tabs for reporting experiments conducted in the four systems described above: bacterial, insect cell, mammalian cell, and cell-free. Fields related to the target, complex, construct, and protein purification are largely consistent across all systems, while expression fields comprise both a shared core and system-specific elements. The metadata also defines the required format for each field, which is generally straightforward, for example, numeric values with units, biological sequences, or free text. The only exception is the reporting of buffers.

The buffer format is described on a separate tab in the spreadsheet. Briefly, we propose recording buffer composition as a semi-structured string beginning with “pH”, followed by the buffer base. The other core components are then listed, each with a defined identity and concentration. The format supports core components of buffer, salt, glycerol, detergent, reducing agent, and ‘other’. The units for concentration are millimolar (mM), %(w/v) or %(v/v) and explicitly recorded in the entry. There can be multiple (or no) instances of a particular component (*e*.*g*., OTHER) in the string. So, for example, a string “pH 7.5; BUFF HEPES, 50 mM; SALT NaCl, 250 mM; GLY 5% (w/v); DET TritonX100, 1% (v/v); RED DTT, 1 mM; OTHER inhibitor, 1 mM; OTHER ATP, 10 mM” describes a buffer with pH = 7.5 based on HEPES, with 250 mM NaCl salt, 5% (w/v) glycerol, 1% (v/v) TritonX100 detergent, 1 mM DTT reducing agent, 1 mM inhibitor and 10 mM adenosine 5′-triphosphate. We envisage the use of defined and searchable chemistry formats (*e*.*g*., CAS Registry Numbers) to aid standardization of data entry.

## Pilot depositions

The proposed data-capture templates were tested on two historical datasets. The subset of the RP3Net training data generated by SGC Stockholm was used as an example of bacterial expression (24). This dataset comprises more than 14,000 data points describing approximately 11,000 constructs. It records the outcomes of small-scale protein expression and purification as categorical readouts (none, low, medium, or high concentration). These outcomes were obtained by manual annotation of relative SDS-PAGE band intensity. The experimental protocol details were curated from the accompanying publication (25).

For mammalian expression, the human secretome dataset was used (26). Human secreted proteins were expressed in Chinese hamster ovary (CHO) and human embryonic kidney (HEK) cells at small, medium, and large scales. Following purification, protein concentrations were measured by spectrophotometry, and quality control was performed by MS. The dataset contains almost 3000 data points describing just over 2000 unique constructs.

The SGC Stockholm dataset was assigned a completeness score of 7/10, while the human secretome dataset received a score of 8/10. The main factor limiting the completeness score of the SGC Stockholm dataset is its reliance on human-annotated categories to describe purification outcomes, rather than on experimentally determined concentration measurements. MS quality control was not performed for this dataset. The human secretome dataset has been assigned an imperfect score because the full vector sequence could not be disclosed due to licensing constraints.

Both datasets were curated to match the proposed format and deposited in BioStudies (https://www.ebi.ac.uk/biostudies/EXPERT/studies/S-EXPD2, https://www.ebi.ac.uk/biostudies/EXPERT/studies/S-EXPD3). We encourage readers of this resource who have access to ongoing experimental data or historical datasets to use these templates for their own depositions.

## Discussion and future opportunities

We outline EXPERT, a roadmap towards data collection to aid predictive protein production centred on the establishment of standardized frameworks for collecting and reporting protein expression and purification data. Community adoption of shared metadata and quality standards is essential to enable interoperability, large-scale data aggregation, and the creation of a sustainable, openly accessible repository. Such infrastructure would provide the foundation for data-driven, generalizable predictive models.

Our primary aim is to initiate a community discussion about which aspects of the protein production process should be captured to facilitate experimental reproducibility and the development of ML models. In its current form, EXPERT represents the outcome of the first stage of this discussion. Looking forward, we envisage that, as EXPERT gains broader community adoption, the templates could form the basis of an intuitive, user-friendly database that encourages community data deposition and supports long-term sustainability. Such a resource could incorporate searchable metadata and sequences (*e*.*g*., using BLAST (27)), automated extraction of data from deposited plasmid sequences or flat files, and a simple interface for uploading individual or batch datasets. The current templates suggest that such a system could be underpinned by a schema comprising entities describing the target, construct, host-dependent expression protocol, purification protocol, and buffers, with the precise structure and relationships defined during software development.

As experimental throughput increases and automation becomes increasingly prevalent, particularly with the emergence of closed-loop laboratories, the framework will also need to support programmatic data capture and access. We therefore envisage that EXPERT could evolve to incorporate industry-standard data formats such as XML or JSON, facilitating automated extraction of metadata directly from laboratory instrumentation and software. This would enable EXPERT to remain compatible with increasingly automated experimental workflows while providing the structured data required for downstream ML model training and evaluation. We hope that use of EXPERT will become as commonplace as UniProt and PDB, both as an entry point for users engaging with their protein targets, but also for bulk data access for AI/ML predictive model development.

Our initial emphasis on bacterial, insect, mammalian, and cell-free systems reflects their widespread use across academic and industrial laboratories. As the dataset matures, expansion to additional hosts will broaden its applicability. In parallel, predictive models should integrate richer molecular descriptors, such as structural predictions, aggregation propensity, proteolytic sensitivity, degradation pathways, and sequence-level indicators of instability or translational stalling. Increasing dataset diversity across constructs, hosts, and experimental conditions will be critical to improving robustness and transferability.

A central challenge in building predictive models for protein expression and purification is defining what constitutes “sufficient” data. This involves not only dataset size but also diversity, quality, and representation. Although large datasets are valuable, volume alone is not sufficient, and incorporation of negative data is essential. Broad sequence diversity and rich construct, expression, and purification metadata used as covariates are critical for model generalization, as models trained on narrow subsets often perform poorly on rare or structurally novel proteins. Evidence from structure prediction shows reduced accuracy for unseen folds or singleton families, highlighting the need for wide coverage across protein classes and organisms.

Another key issue is distinguishing molecular biology-driven determinants of expression from protein-intrinsic determinants of folding and solubility, such as protein class, membrane-spanning or intrinsically disordered regions, post-translational modifications, hydrophobicity, and isoelectric point. High expression levels do not guarantee correct folding or solubility, as unfavorable structural or physicochemical features can impair stability. Predictive frameworks should therefore integrate outputs from structural and biophysical tools, such as structure and disorder prediction models. Clear data inclusion criteria and well-defined model scope will be essential for developing robust, generalizable systems for protein production.

Historical protein production data represent a valuable but underutilized asset. While legacy datasets could substantially expand training data, their integration depends on data completeness, consistency, and metadata quality. Retrospective curation is resource-intensive and only select laboratories or consortia may hold sufficiently standardized archives to justify systematic harmonization. Careful cost-benefit evaluation will therefore be required. Complementary strategies, including automated text mining of experimental methods from resources such as the PDB and the published literature, could further expand structured datasets while minimizing manual burden. Adoption of the framework by editors and publishers could help ‘future-proof’ the literature by promoting more consistent reporting and improving the reproducibility of protein production across laboratories. To achieve this, the framework will need to be clearly defined for this purpose.

Sustained engagement with the ML community will be essential to accelerate progress. Community benchmarking initiatives, potentially coordinated by organisations such as the Structural Genomics Consortium (SGC) (28) or Conscience (https://conscience.ca/beacon/), could establish standardized protein expression and purification challenges to evaluate predictive performance. Analogous efforts in ligand discovery, including CACHE (Critical Assessment of Computational Hit-Finding Experiments) (29), demonstrate how structured challenges can catalyse collaboration and drive methodological advances.

Ultimately, we envision a freely accessible design and search platform that enables researchers to rationally prioritize constructs with higher probabilities of successful expression and purification. By reducing duplication of effort and reliance on empirical screening of many constructs, such a resource would conserve time and resources while accelerating discovery across different protein target classes. This framework represents the first step toward sustainable data harmonization, built on community agreement and the collection of sufficient data to ensure utility, while laying the groundwork for a next-generation searchable database.

## Data availability

Data has been deposited to Biostudies and can be accessed via these links:

https://www.ebi.ac.uk/biostudies/EXPERT/studies/S-EXPD2.

https://www.ebi.ac.uk/biostudies/EXPERT/studies/S-EXPD3

## Supporting information

This article contains supporting information 24, 25, 26, 31, 32

## Conflict of interest

The authors declare that they have no conflicts of interest with the contents of this article.
