# Supplementary material for: EXPERT: A practical and forward-looking guide to capturing protein expression and purification metadata to develop machine learning models
Source: J Biol Chem. 2026 Sep 29;302(11):113610. doi: 10.1016/j.jbc.2026.113610 (PMC13738122; doi:10.1016/j.jbc.2026.113610)
Supplement: Supplementary file 1 [file mmc1.docx]

## **Supplementary Table S1. Existing protein production datasets**

Also see **Figure 1** in the main text.

The following criteria were used for including previously published datasets in this overview:

- The dataset should contain the results of heterologous recombinant protein expression and purification.
- The expressed targets should be fully functional proteins or domains, not short fragments.
- The dataset should cover a significant variety of targets, not just few proteins.

Imbalance between positive and negative outcomes is the percentage of successful outcomes (expressed, produced) in the dataset.

Completeness is defined as the number of metadata fields in Supplementary Sheet S2, that can be inferred from the data and from the accompanying publications. It is a score from 0 to 10, with 0 meaning incomplete, and 10 meaning fully complete.

PSI/TargetTrack reports for each construct the last stage of the protein crystallization pipeline that it has progressed to. However, the reason why the construct has been abandoned is not always reported. This can create confusion between the constructs that have failed to express or produce a viable protein, and those that were abandoned for other reasons (e.g. there being other constructs for the same target, financial reasons, etc.). The entire PSI dataset is largely incomplete, but there are small subsets within it with higher degree of completion. The expression host organism is not reported directly but can sometimes be deduced from the protocol text.

SGC Toronto reports small scale expression testing results for a small portion of the data. The majority of the constructs were expressed in *E. coli*.

**Data References:**

**D1**. Berman, H. M. et al. Protein Structure Initiative – TargetTrack 2000–2017 – all data files. Zenodo <https://doi.org/10.5281/zenodo.821654> (2017).

**D2**. The SGC Consortium. Protein production data from the SGC. BioStudies https://www.ebi.ac.uk/biostudies/SGC/studies/S-BSST681 (2022).
